# Optimizing goat growth and rumen function with monosodium glutamate byproduct-treated rice straw in total mixed rations

**DOI:** 10.14202/vetworld.2025.2427-2438

**Published:** 2025-08-26

**Authors:** Suparada Saphaphan, K. Teepalak Rangubhet, Phongthorn Kongmun

**Affiliations:** Department of Animal Science, Faculty of Agriculture, Kasetsart University, Bangkok, Thailand

**Keywords:** agro-industrial residues, goat fattening, monosodium glutamate by-product, nutrient digestibility, rice straw, rumen fermentation, rumen microbiota, total mixed ration

## Abstract

**Background and Aim:**

Rice straw is a widely available but nutritionally limited roughage for ruminants due to its low protein content and digestibility. This study aimed to evaluate the effects of replacing pangola hay with monosodium glutamate byproduct-treated rice straw (MSGBTRS) in total mixed rations (TMR) on growth performance, nutrient utilization, rumen fermentation, and microbial populations in fattening goats.

**Materials and Methods:**

Sixteen male Anglo-Nubian crossbred goats (3 months old, 15 ± 2 kg) were randomly allocated to four dietary treatments (0%, 25%, 50%, and 75% MSGBTRS replacing pangola hay) in a completely randomized design. MSGBTRS was prepared by blending rice straw with liquid MSGB (8.8:1.2 w/w) and sun-dried. The feeding trial lasted 50 days, followed by a 7-day digestibility study. Feed intake, body weight (BW), rumen fluid, blood, and fecal samples were analyzed. Rumen microbiota was quantified through real-time polymerase chain reaction.

**Results:**

The 25% MSGBTRS group achieved the highest BW gain (6.52 kg; p = 0.034). Dry matter intake declined linearly with increased MSGBTRS levels (p = 0.019). Neutral detergent fiber and acid detergent fiber digestibility were significantly reduced at 75% substitution (p = 0.001). Ruminal ammonia nitrogen concentrations increased with MSGBTRS inclusion, peaking at 75% (19.08 mg/dL; p = 0.029), while blood urea nitrogen remained unaffected. The 25% group exhibited optimal propionate and butyrate levels and a favorable acetate-to-propionate ratio. Total bacterial populations were highest in the 50%–75% groups (p = 0.002), with the greatest *Ruminococcus albus* abundance in the 75% group (p = 0.045).

**Conclusion:**

Substituting pangola hay with 25% MSGBTRS in TMR is optimal for improving growth performance and fiber digestibility in goats, without adverse effects on feed intake or rumen fermentation. MSGBTRS presents a sustainable, cost-effective alternative roughage source, supporting zero-waste livestock production. Future studies should explore long-term impacts on carcass traits, health, and economic viability in larger-scale goat systems.

## INTRODUCTION

In Thailand, the adoption of total mixed ration (TMR) feeding systems for goat fattening offers notable nutritional advantages over traditional feeding methods. TMR ensures a uniform intake of essential nutrients by homogeneously blending forages, concentrates, and supplements, thereby improving feed efficiency, growth performance, and animal health [1]. Incorporating alternative roughage sources into TMR has been shown to enhance both nutritional quality and functional effi-ciency. For instance, adding sweet sorghum and alfalfa to TMR silage improved nutrient digestibility and growth performance in sheep, especially at lower proportions of sweet sorghum, which increased the digestibility of dry matter (DM), crude protein (CP), and neutral detergent fiber (NDF) [2, 3]. In addition, pelleted TMR containing wheat straw has been associated with increased DM intake (DMI) and average daily gain (ADG) compared to conventional TMR forms [4, 5]. Fermented TMR diets that include unconventional roughages further improve nutrient utilization and enhance feed preservation and storage stability [6].

Rice straw is a widely available and cost-effective roughage source in Thailand, especially in regions with limited access to high-quality forages. However, its application is restricted due to its low CP content (typically 4.0%–4.7%) and poor digestibility, primarily resulting from high lignin and silica content in the cell wall matrix [7, 8]. To address these limitations, methods such as bio-fermentation and co-fermentation with probiotics and fibrolytic enzymes have been explored, demonstrating improvements in digestibility, rumen fermentation, and animal performance [9]. Advances in rice breeding have also introduced brittle-stemmed varieties with thinner cell walls, increased hemicellulose and CP content, and reduced cellulose, all of which enhance digestibility in ruminants [10]. These developments support the strategic use of rice straw as an alternative roughage in sustainable feeding systems.

Monosodium glutamate by-product (MSGB) is an organic residue generated during the microbial fermentation process used in the industrial produc-tion of flavor enhancers. This process typically employs carbohydrate-rich substrates such as cassava and molasses. On a DM basis, MSGB contains high organic matter (OM) (894 g/kg), moderate ash (106 g/kg), elevated CP (460 g/kg), and low ether extract (5 g/kg) [11]. MSGB is also rich in free amino acids, particularly glutamate, which serves as a key energy source for rumen microbes. Due to its high protein and amino acid content, MSGB is considered a sustainable and promising alternative feed ingredient in ruminant nutrition.

Experimental studies support its use in enhancing rumen fermentation. Supplementation with MSGB has been shown to increase *in vitro* gas production and volatile fatty acid (VFA) concentrations, indicating improved microbial fermentative activity [12]. When incorporated into rice silage, MSGB improves fermentation quality and enhances acid detergent fiber (ADF) and NDF digestibility. These benefits are primarily due to glutamate’s role as a rapidly fermentable energy source, stimulating microbial growth, enzymatic activity, fiber degradation, and VFA production – key processes for efficient ruminant energy metabolism [13, 14].

MSGB has also been evaluated as an alternative to conventional protein sources such as soybean meal (SBM). Replacing SBM with MSGB in dairy cow diets has been shown to reduce feed costs without compromising milk yield or profitability [11]. Moreover, supplementing goat diets with a mixture of cassava pulp and MSGB (CPMSG) significantly improved CP digestibility and increased ADG [15]. Economically, substituting SBM with MSGB in dairy cow diets can reduce feed costs by 2.9%–17.3%, thereby enhancing overall economic returns [11]. *In vitro* studies have further shown that treating rice straw with MSGB improves its CP content and digestibility, making it a more efficient roughage alternative compared to conventional options like pangola hay [16].

Despite the widespread availability and low cost of rice straw in Thailand, its application as a primary roughage source in small ruminant diets remains limited due to inherent nutritional deficiencies – namely, low CP content and poor digestibility stemming from high lignin and silica levels. Previous research has primarily focused on improving rice straw quality through chemical treatments (e.g., urea or molasses) or microbial fermentation. However, these approaches require extended processing times, additional labor, or pose risks of nutrient losses during ensiling. Furthermore, while MSGB has been evaluated as a supplemental protein source in combination with cassava pulp or in *in vitro* rumen fermentation trials, few studies have investigated its direct application as a treatment agent for rice straw in a TMR system. Most existing studies are either *in vitro* or short-term and fail to examine the integrated effects of MSGB-treated rice straw (MSGBTRS) on growth performance, nutrient utilization, and rumen microbial ecology in small ruminants under practical feeding conditions. In particular, there is a lack of *in vivo* studies assessing graded replacement levels of conventional forages (e.g., pangola hay) with MSGBTRS and their corresponding impact on rumen fermentation dynamics, microbial populations, and systemic nitrogen metabolism in goats.

This study aimed to evaluate the effects of incorporating MSGBTRS into TMRs on the growth performance, feed intake, nutrient digestibility, rumen fermentation characteristics, and rumen microbial populations in fattening goats. By replacing pangola hay with MSGBTRS at incremental levels (0%, 25%, 50%, and 75%), the study sought to identify the optimal substitution rate that maximizes performance without compromising digestibility or rumen health. The experiment employed a comprehensive *in vivo* design, integrating real-time polymerase chain reaction (PCR) analysis of key rumen microbes and monitoring systemic parameters such as ammonia nitrogen and blood urea nitrogen (BUN) levels to provide mechanistic insights into dietary effects. Ultimately, the study aims to offer a sustainable, nutritionally viable, and economically practical feeding strategy that promotes circular agriculture through the use of agro-industrial by-products in small ruminant production systems.

## MATERIALS AND METHODS

### Ethical approval

This study was conducted in accordance with ethical standards and approved by the Institutional Animal Care and Use Committee (Approval ID: ACKU64-AGR-026). All procedures complied with the ARRIVE 2.0 guidelines for *in vivo* animal research.

### Study period and location

The study was conducted from July to December 2022 at the Thepha Livestock Research and Breeding Center, Department of Livestock Development, Songkhla Province, Thailand.

### Experimental design of the experiments

The study followed a completely randomized design with four dietary treatments and four animals per treatment (n = 16). The sample size was determined based on a previous study by Rukboon *et al*. [15], evaluating feed substitution effects in goats, with four animals per group being sufficient to detect significant differences in growth and nutrient utilization parameters under controlled conditions. Animals were stratified by initial body weight (BW) and randomly allocated (Excel RAND function) to four TMR treatments in a completely randomized design. This study investigated the effects of replacing pangola hay with MSGBTRS in TMR at inclusion levels of 0%, 25%, 50%, and 75%. A graded substitution model was employed to evaluate the dose–response relationship and identify the biological threshold at which MSGBTRS could be included without adversely affecting growth performance or rumen function.

### Animals and housing

Sixteen male Anglo-Nubian crossbred goats, approximately 3 months old with an average initial BW of 15 ± 2 kg, were used in the experiment. All animals were housed individually in well-ventilated pens equipped with feeders and automatic watering systems. Mineral-salt blocks were also provided *ad libitum*. The animals were acclimated to the housing and diets for 14 days before the 50-day feeding trial began.

### Diet preparation and feeding management

First, the rice straw was chopped to 2 cm–5 cm lengths to enhance particle homogeneity and mixing efficiency. The chopped material was then blended with liquid MSGB at a ratio of 8.8:1.2 (w/w, straw: MSGB) in a horizontal paddle mixer for 10 min. Immediately after coating, the mixture was sun-dried to lower 13% moisture to stabilize nutrients and prevent microbial spoilage. No ensiling or fermentation step was included because the rapid drying protocol allowed the roughage to be fed without delay, thereby reducing labor and storage time requirements. The dried MSGBTRS was packed in opaque, airtight polyethylene bags, stored under shade, and withdrawn on a first-in, first-out basis for incorporation as the roughage component of the experimental total TMR. Table 1 presents the ingredient composition of the experimental TMR, along with the proximate composition of MSGBTRS and pangola hay. All TMR diets were formulated to contain approximately 16% CP and 68% TDN on a DM basis, in accordance with the nutrient requirements for growing goats as recommended by the National Research Council [17]. Each goat was given a daily feed allowance equivalent to 3.0% of its BW (as-fed basis). All goats underwent a 14-day adaptation period before the experimental trial. Daily feed intake was monitored by weighing the TMR offered and refusals collected, allowing for accurate estimation of actual feed consumption. BW was mea-sured at 14-day intervals to adjust individual feed allowances and track growth performance. The feeding trial was conducted over a period of 50 days following the adaptation phase.

**Table 1:** Feed ingredients and chemical composition of the experimental TMR used in this study.

Ingredient	TMR				MSGBTRS	Pangola hay

	T1 (0%)	T2 (25%)	T3 (50%)	T4 (75%)		
Ingredient (kg DM)						
Pangola hay	60.00	45.00	30.00	15.00		
MSGBTRS	0.00	15.00	30.00	45.00		
Soybean meal	19.50	19.50	19.50	19.50		
Corn	6.00	6.00	6.00	6.00		
Cassava chip	12.50	12.50	12.50	12.50		
Molasses	0.50	0.50	0.50	0.50		
Mineral mix	0.50	0.50	0.50	0.50		
Sulfur	0.50	0.50	0.50	0.50		
Salt	0.50	0.50	0.50	0.50		
Feed price (USD/kg)	0.20	0.19	0.18	0.18		
Chemical composition (g/kg)						
DM	882.30	885.10	885.20	886.30	746.80	953.10
DM (g/kg)						
CP	159.40	162.10	163.80	166.30	84.60	84.20
EE	14.80	8.50	10.30	11.00	25.30	10.90
NDF	603.50	641.70	662.80	682.10	721.50	578.40
ADF	323.20	362.60	379.80	381.90	478.50	348.40

TMR=Total mixed ratio, MSGBTRS=Monosodium glutamate by-product-treated rice straw, DM=Dry matter, CP=Crude protein, EE=Ether extract, NDF=Neutral detergent fiber, ADF=Acid detergent fiber, T1=MSGBTRS replacement at 0%, T2=MSGBTRS replacement at 25%, T3=MSGBTRS replacement at 50%, T4=MSGBTRS replacement at 75%

### Sample collection

The TMR intake was recorded daily by measuring the amount of feed offered and the number of refus-als collected, allowing for accurate calculation of actual feed consumption. A 7-day digestibility trial was conducted following the 50-day feeding trial. During this period, fecal samples were collected once daily by grab sampling directly from the rectum, pooled for each individual goat, oven-dried, ground, and subsequently analyzed for nutrient composition. On the final day of the experiment, rumen fermentation parameters were evaluated using rumen fluid samples collected through stomach tubing at 0, 2, 4, and 6 h after feeding. The collected rumen fluid was filtered through four lay-ers of cheesecloth and divided into two portions for subsequent analyses. The first portion was centrifuged at 16,000 × *g* for 15 min, and the supernatant was stored at −20°C for analysis of ammonia nitrogen (NH_3_–N) and VFA concentrations. The second portion was also stored at −20°C for subsequent DNA extrac-tion to assess microbial populations. A single blood sample was collected through jugular venipuncture during the experiment, concurrent with rumen fluid sampling. Blood samples were collected at 0, 2, 4, and 6 h post-feeding to assess temporal changes in BUN concentration. Immediately after collection, the samples were placed on ice, refrigerated for 1 h, and then centrifuged at 3,500 × *g* for 20 min to separate the plasma. The resulting plasma was stored at −20°C until further analysis.

### Laboratory analyses

#### Feed and fecal composition

Feed and fecal samples were dried to constant weight at 60°C, ground through a 1-mm screen using a Cyclotec mill (Tecator, Sweden) and analyzed for DM and ash content following AOAC standard procedures [18]. CP (calculated as total nitrogen × 6.25) was determined using the Kjeldahl method, as per AOAC guidelines [18]. NDF and ADF were analyzed according to the method of Van Soest *et al*. [19]. All chemical analyses were conducted in duplicate to ensure precision.

#### Rumen fermentation and blood metabolites

Rumen NH_3_–N concentrations were determined using the colorimetric method described by Chaney and Marbach [20]. VFA concentrations were measured using high-performance liquid chromatography equipped with a Waters 600E controller, a Waters 484 ultraviolet (UV) detector (Milford, MA, USA), and a Novapak C18 column (3.9 × 300 mm). The mobile phase used was 10 mmol/L H_2_PO_4_, adjusted to pH 2.5, following the protocol by Samuel *et al*. [21]. BUN concentrations were measured according to the method described by Crocker [22].

### Microbial quantification by real-time PCR

Community DNA was extracted from 1.0 mL aliquots of each sample using the repeated bead beating plus column method [23], which significantly enhances DNA yield. The quality and concentration of DNA were assessed using agarose gel electrophoresis and spectrophotometry. A total of 48 samples were collected across four treatments, 3 time points (0, 2, and 4 h post-feeding), and four replicates for genomic DNA extraction. The DNA concentration was norma-lized before amplification to ensure consistency across samples. For general bacteria (16s ribosomal RNA [rRNA]), the following primers were used for real-time PCR: forward (F) 5′-CGG CAA CGA GCG CAA CCC-3′ and reverse (R) 5′-CCA TTG TAG CAC GTG TGT AGC C-3′, producing a 130-bp product. For general anaerobic fungi (18s rRNA): F 5′-GAG GAA GTA AAA GTC GTA ACA AGG TTT C-3′ and R 5′-CCA TTG TAG CAC GTG TGT AGC C-3′, a 120-bp product was obtained. For general Protozoa (18s rRNA): F 5′-GCT TTC GWT GGT AGT GTA TT-3′ and R 5′-CTT GCC CTC YAA TCG TWC T-3′, a 223-bp product was produced. For *Fibrobacter succinogenes* (16s rRNA): F 5′-GTT CGG AAT TAC TGG GCG TAA A-3′ and R 5′-CGC CTG CCC CTG AAC TAT C-3′, yielding a 121-bp product. *Ruminococcus flavefaciens* primers (16s rRNA), F (5′-CGA ACG GAG ATA ATT TGA TTT ACT TAG G-3′) and R (5′- CGG TCT CTG TAT GTT ATG AGG TAT TAC C-3′) (132-bp product) [24]. For *Ruminococcus albus* (16s rRNA): Ra1281f 5′-CCC TAA AAG CAG TCT TAG TTC G-3′ and Ra1439r 5′-CCT CCT TGC GGT TAG AAC A-3′, yielding a 175-bp product [25].

Six sample-derived DNA standards were prepared from pooled community DNA across the treatments. The sample-derived DNA standards were generated using conventional PCR for each real-time PCR assay. The resulting PCR products were purified using a QIAquick PCR Purification Kit (Qiagen Inc., Valencia, CA, USA) and quantified using a spectrophotometer (SP-UV1100, DLAB). For each standard, the copy number concentration was calculated based on the PCR product length and DNA mass concentration using the following formula:

Copy number (copies/μL) = (6.023 × 10^23^ × [Conc. ng/μL] × 10^-^9)/(BP × 607.4) + 157.9

Where 6.023 × 10^23^ is Avogadro’s number, 607.4 is the average molecular weight of a base pair, and 157.9 is the end modification molecular weight of a DNA molecule.

Tenfold serial dilutions were prepared in tris-ethylenediaminetetraacetic acid buffer before real-time PCR [26]. Six real-time PCR standards were prepared. The conditions for real-time PCR assays were consi-stent with those used in the conventional PCR assays described above. Real-time PCR was performed using a Bio-Rad CFX Maestro 1.1 thermal cycler (Bio-Rad Laboratories, Hercules, CA, USA). Each reaction was conducted in a final volume of 20 μL, including 10 μL of SYBR Green Master Mix, 0.5 μL of each primer (10 μM), 2 μL of template DNA, and 7 μL of nuclease-free water. The cycling protocols were described by Makkar and McSweeney [24] and Koike and Kobayashi [25]. All PCR assays were performed in duplicate.

### Statistical analysis

All data were analyzed using Statistical Analysis System (SAS) software version 9.4 (SAS Institute Inc., Cary, NC, USA). Before the analysis, the normality and homogeneity of variances were verified using the Shapiro–Wilk test. Data were subjected to one-way analysis of variance using the GLM procedure, with the following statistical model:

Y_ij_ = μ + T_i_ + ε_ij_

where Y_ij_ is the observation from treatment i and replicate j; μ is the overall mean, T_i_ is the treatment effect; and ε_ij_ is the residual error. When significant differences were detected (p < 0.05), the means were separated using Duncan’s new multiple range test [27]. Orthogonal polynomial contrasts were performed to evaluate the dose-response trends associated with the increasing inclusion levels of MSGBTRS. Statistical significance was declared at p < 0.05, while 0.05 ≤ p < 0.10 was considered statistically significant.

## RESULTS

### Chemical composition of experimental diets

Table 1 presents the chemical composition of the roughage sources used in this study. Mixing RS with MSGB at an 8.8:1.2 (w/w) ratio increased the CP content to 84.60 g/kg (8.46%), which is comparable to the CP level of pangola hay (84.20 g/kg) used in this experiment. In this study, we replaced pangola hay with MSGBTRS at inclusion levels of 0%, 25%, 50%, and 75% to assess its potential as a roughage source in TMR. All four TMR formulations had similar CP concentrations, ranging from 159.40 to 166.30 g/kg, thereby meeting the dietary protein requirements across treatments. These formulations were deemed suitable for evaluating the effects of the inclusion of MSGBTRS on growth performance and nutrient use in fattening goats.

### Growth performance and feed intake

As presented in Table 2, replacing pangola hay with MSGBTRS had a significant linear effect on final BW, total weight gain, and ADG in goats (p = 0.012, p = 0.011, and p = 0.016, respectively). Increasing MSGBTRS inclusion levels were associated with a significant decline in both weight gain and ADG. Notably, goats receiving the 25% MSGBTRS diet showed the greatest BW gain (6.52 kg; p = 0.034). In contrast, the feed conversion ratio was not significantly influenced by the MSGBTRS substitution (p > 0.05). Feed intake showed a significant linear decrease as MSGBTRS levels increased. Specifically, DMI (g/day and g/kg BW/day) and CP intake decreased significantly with increasing MSGBTRS levels (p = 0.019, p = 0.034, and p = 0.036, respectively).

**Table 2:** Effects of substituting pangola hay with MSGBTRS on feed consumption, growth performance, and nutrient digestibility.

Item	Level of MSGBTRS replacement (%)				SEM	p-value	Orthogonal polynomial		
	
	T1 (0%)	T2 (25%)	T3 (50%)	T4 (75%)			lin	qua	cub
Initial weight (kg)	16.52	16.55	16.66	16.67	0.590	0.999	0.925	0.996	0.975
Final weight (kg)	22.30	23.07	21.18	20.01	0.660	0.348	0.012	0.167	0.254
BW gain (kg)	5.77^b^	6.52^a^	4.52^b^	3.28^b^	0.430	0.034	0.011	0.171	0.259
ADG (g/day)	0.10	0.11	0.08	0.05	0.007	0.055	0.016	0.119	0.397
FCR	7.97	6.36	7.80	10.38	0.280	0.326	0.205	0.162	0.759
Feed intake									
DM (g/day)	638.20	606.60	493.70	459.60	29.960	0.094	0.019	0.981	0.487
DM (g/BW/day)	28.62	26.18	23.63	22.59	1.020	0.160	0.034	0.713	0.842
CP (g/day)	101.70	98.32	80.84	76.45	4.686	0.150	0.036	0.953	0.473
NDF (g/day)	339.70	355.80	298.10	261.40	16.040	0.179	0.054	0.389	0.472
ADF (g/day)	182.00	194.70	167.10	155.50	8.350	0.412	0.181	0.466	0.462
Digestibility, g/100g of DM									
DM	53.02	55.49	52.35	51.87	0.810	0.435	0.267	0.387	0.267
OM	84.95^a^	82.72^ab^	78.64^bc^	76.55^c^	0.955	0.001	0.001	0.943	0.368
CP	66.85	67.27	66.86	67.63	0.850	0.991	0.850	0.993	0.820
NDF	68.73^a^	65.23^a^	61.70^a^	50.40^b^	1.930	0.001	0.001	0.087	0.491
ADF	53.22^a^	55.60^a^	47.46^a^	39.04^b^	2.010	0.001	0.001	0.072	0.301

^a–c^Means with different superscripts in row are significantly different (p *<* 0.05), SEM=Standard error of the mean, MSGBTRS=Monosodium glutamate by-product treated rice straw, BW=Body weight, ADG=Average daily gain, FCR=Feed conversion ratio, DM=Dry matter, CP=Crude protein, NDF=Neutral detergent fiber, ADF=Acid detergent fiber, T1=MSGBTRS replacement at 0%, T2=MSGBTRS replacement at 25%, T3=MSGBTRS replacement at 50%, T4=MSGBTRS replacement at 75%

### Nutrient digestibility

Moreover, increasing MSGBTRS inclusion resulted in a significant reduction in the digestibility of OM, NDF, and ADF (p = 0.001). OM digestibility was highest in the 0% substitution group (84.95 g/100g of DM; p = 0.001) and was not significantly different from the 25% group (82.72 g/100g of DM; p > 0.05). Similarly, NDF and ADF digestibility were highest in the 0%, 25%, and 50% substitution groups, with a significant decline observed at 75% inclusion (p = 0.001).

### Rumen ammonia and BUN

Increasing the MSGBTRS substitution level for pangola hay resulted in a significant linear increase in ruminal NH_3_–N concentration (p < 0.05; Table 3). Specifically, NH_3_–N concentrations at 2 and 6 h post-feeding and the mean values increased linearly with higher MSGBTRS inclusion (p = 0.012, p = 0.003, and p = 0.008, respectively). The highest mean NH_3_–N concentration occurred in the 75% substitution group (19.08 mg/dL), whereas the lowest was found in the 0% substitution group (12.16 mg/dL; p = 0.029). In contrast, MSGBTRS inclusion did not significantly influence BUN levels (p > 0.05).

**Table 3:** Effects of substituting pangola hay with MSGBTRS on ammonia nitrogen concentration and BUN.

Item	Level of MSGBTRS replacement (%)				SEM	p-value	Orthogonal polynomial		
	
	T1 (0%)	T2 (25%)	T3 (50%)	T4 (75%)			lin	qua	cub
NH_3_-N (mg/dL)									
0 h	11.34	12.34	10.69	16.36	1.200	0.365	0.228	0.343	0.364
2 h	15.23	19.50	20.82	21.54	0.939	0.055	0.012	0.272	0.739
4 h	11.92	16.15	14.79	18.02	1.130	0.299	0.110	0.821	0.319
6 h	10.14^b^	14.01^ab^	11.97^b^	20.41^a^	1.270	0.007	0.003	0.221	0.060
Mean	12.16^b^	15.50^ab^	14.57^ab^	19.08^a^	0.890	0.029	0.008	0.684	0.145
BUN (mg/dL)									
0 h	25.57	25.90	27.63	23.65	1.210	0.759	0.733	0.423	0.551
2 h	28.22	29.22	25.90	25.25	0.750	0.203	0.075	0.568	0.288
4 h	28.45	28.50	26.75	25.72	0.730	0.504	0.163	0.725	0.712
6 h	26.70	28.02	25.40	24.00	0.940	0.520	0.239	0.495	0.561
Mean	27.22	27.92	26.42	24.70	0.730	0.478	0.196	0.428	0.770

^a,b^Means with different superscripts in row are significantly different (p *<* 0.05), SEM=Standard error of the mean, MSGBTRS=Monosodium glutamate by-product treated rice straw, BUN=Blood urea nitrogen, T1=MSGBTRS replacement at 0%, T2=MSGBTRS replacement at 25%, T3=MSGBTRS replacement at 50%, T4=MSGBTRS replacement at 75%

### VFA profiles

Replacing pangola hay with MSGBTRS significantly affected ruminal VFA production (p < 0.05; Table 4). At 0 h post-feeding, the total VFA concentration increased linearly with higher levels of MSGBTRS inclusion (p = 0.033). Although total VFA production also tended to increase at 6 h post-feeding and for the overall mean, these changes were not statistically significant (p = 0.091 and p = 0.081, respectively). A consistent linear increase in the acetate molar proportion was observed with higher levels of MSGBTRS (p < 0.05). The 75% substitution group exhibited the highest acetate proportions at 2 and 4 h and in the overall mean (p = 0.001, p = 0.025, and p = 0.016, respectively). In contrast, propionate concentrations declined linearly with increasing MSGBTRS levels at 2 h post-feeding and in the mean values (p = 0.007 and p = 0.040, respectively). The highest propionate proportion was observed in the 25% substitution group at 2 h post-feeding (23.44 mol/100 mol total VFAs; p = 0.027). In addition, butyrate molar proportion declined linearly with increasing MSGBTRS inclusion at 2 h, 4 h, and in the overall mean (p = 0.002, p = 0.022, and p = 0.036, respectively), with the highest butyrate value observed in the 0% group at 2 h (10.71 mol/100 mol total VFAs; p = 0.017). The acetate-to-propionate (C2:C3) ratio increased significantly with increasing levels of MSGBTRS (p < 0.05), with the lowest ratio recorded in the 25% substitution group (p = 0.014).

**Table 4:** Effects of substituting pangola hay with MSGBTRS on the concentration of volatile fatty acids.

Item	Level of MSGBTRS replacement (%)				SEM	p-value	Orthogonal polynomial		
	
	T1 (0%)	T2 (25%)	T3 (50%)	T4 (75%)			lin	qua	cub
Total VFAs (mmol/L)									
0 h	37.88	46.47	41.18	61.15	3.530	0.076	0.033	0.362	0.170
2 h	59.37	70.36	74.58	65.30	2.730	0.238	0.359	0.073	0.775
4 h	56.34	62.73	59.43	59.51	2.200	0.824	0.775	0.520	0.549
6 h	50.94	59.41	59.39	63.40	2.340	0.310	0.091	0.632	0.549
Mean	51.13	59.74	58.64	62.34	2.010	0.243	0.081	0.532	0.412
Acetate (mol/100 mol total VFAs)									
0 h	71.46	71.45	72.59	74.78	0.570	0.121	0.031	0.301	0.982
2 h	66.60^b^	67.43^b^	72.01^a^	74.40^a^	0.990	0.001	0.001	0.531	0.295
4 h	68.71^b^	68.08^b^	71.89^ab^	73.68^a^	0.810	0.025	0.006	0.356	0.273
6 h	70.71	69.37	72.66	74.42	0.780	0.093	0.034	0.273	0.328
Mean	69.37^b^	69.08^b^	72.29^ab^	74.32^a^	0.740	0.016	0.003	0.314	0.364
Propionate (mol/100 mol of total VFAs)									
0 h	20.15	20.33	19.99	18.57	0.460	0.527	0.262	0.424	0.896
2 h	22.68^ab^	23.44^a^	19.66^bc^	18.54^c^	0.720	0.027	0.007	0.422	0.180
4 h	20.92	21.91	19.74	18.83	0.500	0.141	0.059	0.315	0.298
6 h	20.16	21.16	19.33	18.47	0.440	0.170	0.083	0.276	0.315
Mean	20.98	21.71	19.68	18.60	0.500	0.118	0.040	0.328	0.370
Butyrate (mol/100 mol of total VFAs)									
0 h	8.37	8.20	7.40	6.65	0.460	0.681	0.211	0.777	0.884
2 h	10.71^a^	9.13^ab^	8.32^b^	7.05^b^	0.450	0.017	0.002	0.822	0.695
4 h	10.36	10.00	8.36	7.48	0.490	0.119	0.022	0.777	0.611
6 h	9.12	9.46	8.00	7.09	0.050	0.346	0.111	0.536	0.604
Mean	9.64	9.20	8.02	7.07	0.450	0.182	0.036	0.767	0.803
Acetate: Propionate (mol/100 mol of total VFAs)									
0 h	3.57	3.53	3.66	4.08	0.110	0.289	0.107	0.303	0.894
2 h	2.96^bc^	2.91^c^	3.71^ab^	4.05^a^	0.160	0.014	0.002	0.436	0.259
4 h	3.31	3.13	3.67	3.94	0.120	0.062	0.020	0.282	0.296
6 h	3.53	3.30	3.77	4.04	0.110	0.103	0.044	0.229	0.323
Mean	3.34	3.21	3.70	4.03	0.120	0.053	0.014	0.276	0.402

^a–c^Means with different superscripts in row are significantly different (p < 0.05), SEM=Standard error of the mean, MSGBTRS=Monosodium glutamate by-product treated rice straw, T1=MSGBTRS replacement at 0%, T2=MSGBTRS replacement at 25%, T3=MSGBTRS replacement at 50%, T4=MSGBTRS replacement at 75%

### Rumen microbial populations

Regarding rumen microbiota, the total bacterial populations at 2 h post-feeding increased significantly with higher MSGBTRS inclusion (p = 0.001), reac-hing peak values in the 50%–75% substitution groups (11.05–11.54 × 10^12^ cell/mL; p = 0.002) (Table 5). However, the substitution of MSGBTRS had no significant effect on the populations of ruminal fungi, Protozoa, *F. succinogenes*, or *R. flavefaciens* (p > 0.05). The *R. albus* population showed both linear and quadratic responses in 4 h post-feeding and in mean values (p = 0.005 and p = 0.015, respectively). The highest mean abundance of *R. albus* was observed in the 75% MSGBTRS group (3.49 × 10^4^ cell/mL; p = 0.045).

**Table 5:** Effects of substituting pangola hay with MSGBTRS on rumen microorganism populations.

Item	Level of MSGBTRS replacement (%)				SEM	p-value	Orthogonal polynomial		
	
	T1 (0%)	T2 (25%)	T3 (50%)	T4 (75%)			lin	qua	cub
Bacteria (×10^12^ cell/mL)									
0 h	1.47	4.50	2.87	4.93	0.680	0.266	0.157	0.716	0.176
2 h	2.34^b^	6.15^b^	11.54^a^	11.05^a^	1.180	0.002	0.001	0.178	0.288
4 h	9.17	15.5	31.46	5.75	6.430	0.546	0.925	0.247	0.404
Mean	6.12	1.74	1.48	3.12	2.390	0.456	0.490	0.219	0.450
Fungi (×10^7^ cell/mL)									
0 h	0.20	2.62	1.50	2.94	0.680	0.557	0.295	0.744	0.364
2 h	8.10	0.76	10.60	13.00	4.060	0.848	0.530	0.618	0.602
4 h	0.23	3.78	0.50	2.53	1.130	0.751	0.765	0.799	0.420
Mean	6.95	3.20	8.50	6.18	2.460	0.901	0.893	0.875	0.482
*Protozoa* (×10^7^ cell/mL)									
0 h	0.76	1.97	1.41	1.13	0.270	0.512	0.827	0.212	0.434
2 h	7.69	3.75	4.97	8.93	1.190	0.420	0.648	0.122	0.823
4 h	1.85	1.78	2.13	1.95	2.780	0.980	0.818	0.931	0.740
Mean	3.43	2.50	2.84	4.01	0.390	0.583	0.580	0.218	0.906
*Fibrobacter succinogenes* (×10^7^ cell/mL)									
0 h	0.85	0.75	0.15	0.28	0.150	0.352	0.136	0.079	0.389
2 h	1.29	1.24	6.74	1.30	1.010	0.276	0.511	0.203	0.114
4 h	1.15	0.83	0.80	1.77	0.190	0.475	0.383	0.156	0.679
Mean	1.02	0.89	1.55	1.17	0.290	0.894	0.710	0.844	0.532
*Ruminococcus albus* (×10^4^ cell/mL)									
0 h	0.69	2.08	0.19	2.40	0.640	0.611	0.604	0.765	0.243
2 h	1.71	1.69	1.01	6.60	1.030	0.195	0.127	0.168	0.432
4 h	5.19^a^	1.80^b^	1.29^b^	0.39^b^	0.630	0.019	0.005	0.211	0.441
Mean	2.53^b^	1.86^ab^	0.82^c^	3.49^a^	0.360	0.045	0.494	0.015	0.149
*Ruminococcus flavefaciens* (×10^5^ cell/mL)									
0 h	1.62	0.92	4.09	1.52	0.550	0.187	0.540	0.379	0.057
2 h	1.58	2.08	3.42	2.13	0.310	0.201	0.277	0.151	0.210
4 h	2.54	2.46	12.1	1.03	2.640	0.462	0.833	0.322	0.225
Mean	1.91	1.82	6.53	1.56	0.940	0.190	0.649	0.189	0.089

^a,b,c^Means with different superscripts in row are significantly different (p < 0.05), SEM=Standard error of the mean, MSGBTRS=Monosodium glutamate by-product treated rice straw, T1=MSGBTRS replacement at 0%, T2=MSGBTRS replacement at 25%, T3=MSGBTRS replacement at 50%, T4=MSGBTRS replacement at 75%

## DISCUSSION

### Composition and nutritional potential of MSGBTRS

This study demonstrates that MSGB can effec-tively increase the protein content of rice straw and other low-protein roughages. In this study, rice straw was enriched with MSGB to match the CP content of pangola hay, allowing it to serve as a comparable roughage source in TMR formulations. Previous studies have demonstrated the use of MSGB as a protein source in ruminant diets, including its substitution for SBM in dairy cows [11], its combination with cassava pulp in goat fattening diets [15], and its enhancement of rice straw protein content compared to urea-treated rice straw and pangola hay [16, 28]. Notably, fermenting rice straw with MSGB for 7–14 days has been reported to not significantly alter the chemical composition of the MSGBTRS product [28].

In this study, MSGBTRS was directly incorporated into TMR without prior fermentation. Unlike previ-ous approaches involving fermentation, this method streamlines on-farm feed preparation while preserving soluble protein and amino acids that may be degraded during fermentation. Thus, the direct use of MSGBTRS offers a practical and nutritionally efficient strategy for using agro-industrial residues in ruminant diets.

### Feed intake and growth performance

The highest weight gain was achieved in goats receiving 25% MSGBTRS, whereas higher inclusion levels (50%–75%) led to reduced performance. This decline in performance is attributed to the corre-sponding reduction in DM and CP intake in goats fed with higher levels of MSGBTRS. The lower feed consumption was accompanied by reduced OM, NDF, and ADF digestibility, likely due to compositional and fiber structure differences between pangola hay and rice straw.

Pangola hay generally contains more CP and has a more balanced nutrient profile, which enhances its palatability and digestibility. These characteris-tics support higher voluntary intake in ruminants. In contrast, rice straw has low CP content and high levels of lignin and silica – structural components that are difficult to digest and adversely affect both nutritional value and palatability [29]. Elevated NDF and ADF levels in rice straw reduce its digestibility and increase its bulk density, limiting intake due to greater rumen fill and the higher energy demand for fiber breakdown [30]. The coarse texture and unappealing flavor of rice straw further reduce its palatability and, consequently, its intake.

Although ensiling with urea or molasses has improved the nutritional quality and acceptability of rice straw [31], this study employed a direct, non-fermented mixture of MSGB and rice straw. This approach effectively increased the CP content of rice straw, enabling it to replace up to 25% of pangola hay in the TMR without negatively impacting intake or performance.

Increasing the protein content of rice straw is a key to improving its palatability and digestibility. For example, McCann [32] found that supplementing rice straw with protein-rich sources, such as cottonseed meal or dried distillers’ grains, increased the intake of OM and total digestible OM in cattle, resulting in a linear increase in forage consumption. These findings support the conclusion that increasing protein availability in low-quality forages can enhance voluntary intake and improve nutrient use in ruminants.

### Rumen fermentation parameters

Replacing pangola hay with higher levels of MSGBTRS led to increased ruminal NH_3_–N concentra-tions, likely due to the greater availability of rapidly degradable nitrogen from MSGB. However, the increase in NH_3_–N did not correspond with significant changes in BUN levels. MSGB is a by-product of micro-bial fermentation used in monosodium glutamate production, commonly using starchy or sugary sub-strates. Consequently, MSGB contains nitrogenous compounds that are readily fermentable by ruminal microbes, resulting in rapid NH_3_–N release upon ingestion.

Similar results have been reported in previous studies. Padunglerk *et al*. [11] observed in lactating Holstein cows that replacing SBM with MSGB at 20%–60% increased ruminal NH_3_–N concentrations, with the lowest levels occurring at the 20% substitution rate. Similarly, Rukboon *et al*. [15] found that supplementing goat diets with 10% and 15% cassava pulp–MSGB mixtures significantly elevated NH_3_–N concentrations at the higher level, indicating that moderate MSGB inclusion may improve nitrogen utilization without cau-sing excessive ammonia accumulation.

Although NH_3_–N and BUN are typically correlated, the absence of significant differences in BUN among treatments suggests that nitrogen from MSGBTRS was used as efficiently as that from pangola hay. This implies that the NH_3_–N generated was efficiently incorporated into microbial protein rather than being converted to urea and excreted. Ruminants can utilize non-protein nitrogen sources, such as urea, by converting ammonia into microbial protein in the rumen, which constitutes a major metabolizable protein component [33]. However, efficient nitrogen use requires synchronization between the availability of fermentable energy and rumen-degradable protein. An imbalance can lead to excess NH_3_–N, which is detoxified in the liver and excreted as urea, resulting in nitrogen loss [34].

In this study, similar BUN levels across treat-ments indicate that protein from MSGBTRS was metabolically used as efficiently as pangola hay, supporting its viability as an alternative nitrogen source in ruminant diets.

Replacing pangola hay with increasing levels of MSGBTRS significantly elevated acetate production, while the 25% substitution level resulted in optimal propionate and butyrate concentrations and a desirable acetate-to-propionate (C2:C3) ratio. These shifts in the VFA profile are likely due to the higher proportion of rice straw in the diet at higher MSGBTRS inclusion levels.

Rice straw is naturally high in structural carbo-hydrates, such as cellulose and hemicellulose, which slow fermentation and extend the rumen retention time, conditions that promote acetate production by fibrolytic microbes [35]. The coarse texture and larger particle size of rice straw also help maintain a stable rumen environment, supporting cellulolytic microbial activity and increasing acetate production [36]. In contrast, the fine particle size and higher nutrient density of pangola hay may lead to less rumen stability and support microbial populations that favor propionate and butyrate production [35].

The rumen microbiome composition plays a critical role in determining the VFA profile. Fibrous feedstuffs, such as rice straw, are primarily degraded by cellulolytic and hemicellulolytic bacteria, such as *F. succinogenes* and *R. albus*, which are efficient producers of acetate [35]. Conversely, the more fermentable fractions of pangola hay promote non-cellulolytic microbes that generate higher propionate and butyrate levels.

Supplementing diets with nitrogen sources, such as urea or protein-rich ingredients, such as SBM, can alter rumen fermentation by boosting microbial prot-ein synthesis and encouraging propionate-producing bacterial growth. Such shifts may lower the C2:C3 ratio and improve the efficiency of energy utilization in ruminants [37]. In this study, the 25% MSGBTRS level likely offered a balanced supply of structural fiber and fermentable nitrogen, creating favorable conditions for both acetate and propionate production in the rumen.

### Rumen microbial dynamics

A significant increase in the total bacterial population was observed at 2 h post-feeding with hig-her levels of MSGBTRS inclusion, with the greatest abundance recorded in the 50%–75% substitution groups. This finding suggests that MSGBTRS promotes ruminal bacterial growth during the early postprandial phase, likely due to its favorable nutrient profile and fermentability.

The high CP content of MSGB supports micro-bial protein synthesis, which is essential for sustaining bacterial proliferation in the rumen. Compared with conventional non-protein nitrogen sources, such as urea, MSGB tends to result in lower ruminal NH_3_–N concentrations, indicating more efficient nitrogen use by rumen microbes [38]. This improved efficiency may be attributed to the presence of readily assimilable amino acids and peptides in MSGB, which enhances microbial growth without accumulating excess ammonia.

Several studies have demonstrated that MSGB supplementation can modulate the rumen microbiota by increasing the relative abundances of beneficial bacterial genera, such as *Prevotella, Ruminococcus*, and *Butyrivibrio*, while simultaneously reducing the prevalence of less desirable microbial populations [39]. These microbial composition shifts are conducive to improved fiber degradation and fermentation efficiency.

The *R. albus* population exhibited both linear and quadratic trends over time, with the most pronounced increase observed in the group receiving 75% MSGBTRS substitution. Given the pivotal role of *R. albus* in cellulose degradation, this growth pattern suggests that higher MSGBTRS inclusion levels may enhance fiber degradation efficiency in the rumen.

*R. albus* secretes a range of cellulolytic enzymes that hydrolyze cellulose into readily fermentable substrates, thereby facilitating microbial metabolism in the rumen [40]. *R. albus* can significantly enhance fiber digestibility, particularly when cocultured with non-fibrolytic bacteria such as *Selenomonas ruminantium*. Such microbial interactions improve the digestibility of fibrous feedstuffs, including rice straw and para grass, and increase VFA production, which plays a central role in ruminant energy metabolism [41].

In the context of this study, the observed increase in the *R. albus* population at higher MSGBTRS inclusion levels may indicate that MSGBTRS provides an optimal rumen environment – potentially through improved fiber structure, stabilized rumen pH, or the availability of specific nutrients that favor cellulolytic bacterial growth. This interpretation is consistent with the concurrent rise in acetate production observed at the 75% MSGBTRS substitution level, as acetate is a prim-ary fermentation end-product of cellulolytic bacterial metabolism.

### Integrative interpretation and study significance

This study offers a comprehensive physiological assessment of MSGBTRS use in goats, going beyond conventional performance metrics. By integrating data on growth performance, nutrient digestibility, rumen fermentation end-products, microbial populations, and BUN, the study provides a holistic view of metabolic responses to graded MSGBTRS levels.

This level of integration is rarely reported in short-term goat trials and sets this research apart from previous MSGB-related studies that typically focused on single or limited parameters. Such an approach enhances the understanding of both nutritional value and rumen microbial adaptation, supporting the practical application of MSGBTRS in small ruminant production systems.

## CONCLUSION

This study evaluated the feasibility of replacing pangola hay with MSGBTRS in TMR diets for fattening goats. The results demonstrated that MSGBTRS is a nutritionally viable roughage alternative, particu-larly at moderate inclusion levels. Replacing pangola hay with MSGBTRS at 25% resulted in the highest BW gain (6.52 kg), optimal ADG, and maintained DMI and nutrient digestibility. In contrast, higher inclusion levels (50%–75%) led to a significant reduction in performance, DMI, and digestibility of OM, NDF, and ADF. Ruminal ammonia nitrogen and acetate concentrations increa-sed significantly with higher MSGBTRS levels, whereas propionate and butyrate concentrations declined. The total bacterial population, including *R. albus*, also increased in goats receiving higher MSGBTRS levels, indicating microbial adaptation and enhanced fiber-degrading activity.

Incorporating MSGBTRS at a 25% substitution level offers a practical, cost-effective strategy for reducing dependence on conventional forages such as pangola hay while supporting sustainable livestock production. The use of agro-industrial residues like MSGB in ruminant diets also contributes to waste valorization and environmental sustainability. A key strength of this study lies in its comprehensive approach, integrating animal performance, nutrient digestibility, fermentation characteristics, and microbial dynamics to evaluate the physiological impact of MSGBTRS.

However, the study was limited by its short-term duration, single goat breed, and specific housing conditions. It also did not compare the tested direct mixing method with other processing techniques such as ensiling or pelleting. Future research should assess the long-term impacts of MSGBTRS on carcass quality, nitrogen excretion, and microbial ecology using advanced techniques such as metagenomics or metabolomics.

In conclusion, MSGBTRS can effectively replace up to 25% of pangola hay in goat diets without com-promising growth or nutrient utilization, offering a promising strategy for enhancing feed resource sustai-nability and economic efficiency in small ruminant pro-duction systems.

## AUTHORS’ CONTRIBUTIONS

PK: Conceptualized and designed the study, sup-ervised the overall project, and critically revised the manuscript. SS: Conducted the experiments, managed data collection and analysis, and drafted the initial manuscript. KR: Sample collection and laboratory ana-lyses. All authors have read and approved the final version of the manuscript.
